# 2-(3-Meth­oxy­phen­oxy)pyrimidine

**DOI:** 10.1107/S160053681003014X

**Published:** 2010-07-31

**Authors:** Shah Bakhtiar Nasir, Zanariah Abdullah, Zainal A. Fairuz, Seik Weng Ng, Edward R. T. Tiekink

**Affiliations:** a Department of Chemistry, University of Malaya, 50603 Kuala Lumpur, Malaysia

## Abstract

In the title compound, C_11_H_10_N_2_O_2_, the benzene ring faces towards one of the pyrimidine N atoms, and is almost orthogonal to the plane through the pyrimidine ring [dihedral angle = 84.40 (14)°]. In the crystal, the presence of C—H⋯π and π–π [centroid–centroid separation = 3.7658 (18) Å] inter­actions leads to a supra­molecular array in the *ac* plane. The layers thus formed inter­digitate along the *b* axis.

## Related literature

For background to the fluorescence properties of compounds related to the title compound, see: Kawai *et al.* (2001 ▶); Abdullah (2005 ▶).
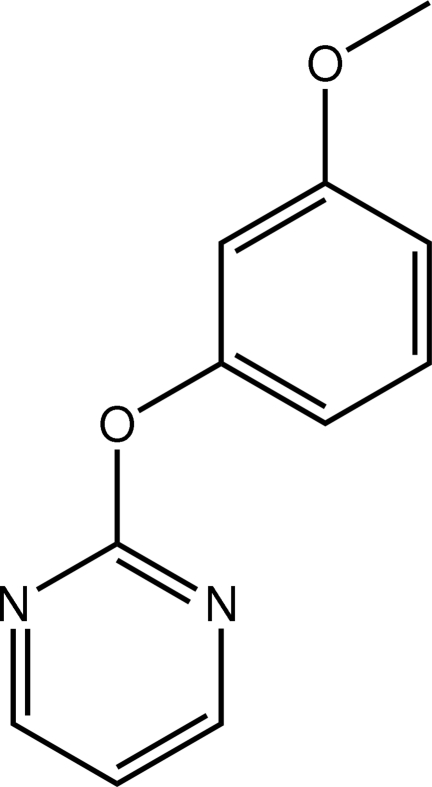


## Experimental

### 

#### Crystal data

C_11_H_10_N_2_O_2_
*M* *_r_* = 202.21
Monoclinic, 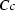

*a* = 8.8120 (16) Å
*b* = 18.215 (3) Å
*c* = 7.2094 (10) Å
β = 119.380 (2)°
*V* = 1008.4 (3) Å^3^
*Z* = 4
Mo *K*α radiation
μ = 0.09 mm^−1^
*T* = 293 K
0.40 × 0.30 × 0.08 mm

#### Data collection

Bruker SMART APEX CCD diffractometer
Absorption correction: multi-scan (*SADABS*; Sheldrick, 1996 ▶) *T*_min_ = 0.889, *T*_max_ = 1.000
4725 measured reflections
1165 independent reflections
897 reflections with *I* > 2σ(*I*)
*R*_int_ = 0.033

#### Refinement

*R*[*F* ^2^ > 2σ(*F* ^2^)] = 0.032
*wR*(*F* ^2^) = 0.086
*S* = 1.02
1165 reflections
138 parameters
2 restraints
H-atom parameters constrained
Δρ_max_ = 0.10 e Å^−3^
Δρ_min_ = −0.10 e Å^−3^
Absolute structure: nd

### 

Data collection: *APEX2* (Bruker, 2009 ▶); cell refinement: *SAINT* (Bruker, 2009 ▶); data reduction: *SAINT*; program(s) used to solve structure: *SHELXS97* (Sheldrick, 2008 ▶); program(s) used to refine structure: *SHELXL97* (Sheldrick, 2008 ▶); molecular graphics: *ORTEP-3* (Farrugia, 1997 ▶) and *DIAMOND* (Brandenburg, 2006 ▶); software used to prepare material for publication: *publCIF* (Westrip, 2010 ▶).

## Supplementary Material

Crystal structure: contains datablocks global, I. DOI: 10.1107/S160053681003014X/hb5584sup1.cif

Structure factors: contains datablocks I. DOI: 10.1107/S160053681003014X/hb5584Isup2.hkl

Additional supplementary materials:  crystallographic information; 3D view; checkCIF report

## Figures and Tables

**Table 1:** Hydrogen-bond geometry (Å, °) *Cg*2 is the centroid of the C5–C10 ring.

*D*—H⋯*A*	*D*—H	H⋯*A*	*D*⋯*A*	*D*—H⋯*A*
C4—H4⋯*Cg*2^i^	0.93	2.89	3.710 (4)	148

Symmetry code: (i) 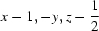.

**Fig. 1.**
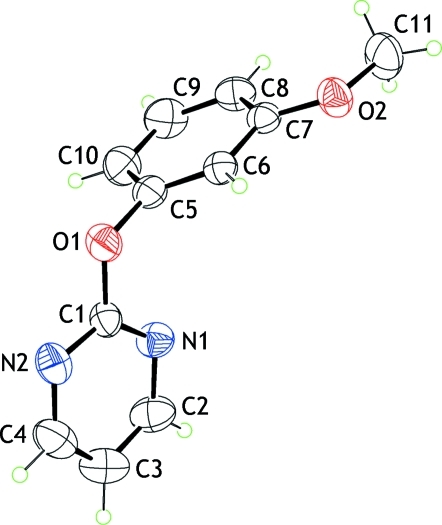
The molecular structure of (I) showing displacement ellipsoids at the 35% probability level.

**Fig. 2.**
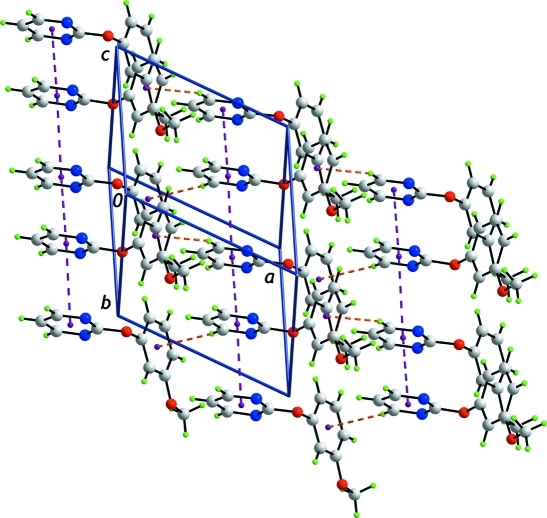
Supramolecular layer in (I) mediated by C–H···π and π–π interactions, shown as orange and purple dashed lines, respectively.

**Fig. 3.**
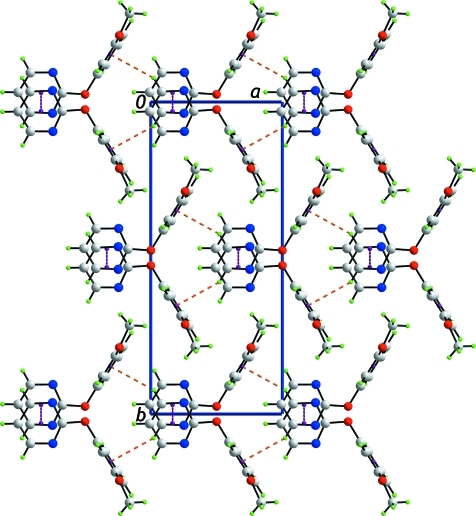
Unit-cell contents shown in projection down the c axis in (I), highlighting the stacking of layers. The C–H···π and π–π interactions are shown as orange and purple dashed lines, respectively.

## supplementary crystallographic information

### Comment

Interest in the title compound stems from interesting fluorescence properties of related compounds (Kawai *et al.* 2001; Abdullah, 2005). In (I), the least-squares plane through the pyrimidine ring bisects the plane through the benzene ring with the C5 and C8 atoms of the latter lying in the plane; the dihedral angle between the planes is 84.40 (14) °. The benzene ring lies to one side of the pyrimidine ring, being proximate to the N1 atom. The methoxy group is almost co-planar with the benzene ring to which is bonded as seen in the value of the C11–O2–C7–C6 torsion angle of 171.7 (2) °.

In the crystal, the presence of C–H···π interactions, formed between pyrimidine-H atoms and benzene rings, and π–π interactions [centroid-centroid separation = 3.7658 (18)Å], formed between pyrimidine rings, leads to the formation of layers in the *ac* plane, Fig. 2 and Table 1. Layers comprise alternating rows of pyrimidine and benzene molecules, and inter-digitate along the *b* axis as shown in Fig. 3.

### Experimental

3-Methoxyphenol (2.2 ml, 20 mmol) was mixed with sodium hydroxide (0.8 g, 20 mmol) in several drops of water. The water was then evaporated. The paste was heated with 2-chloropyrimidine (2.3 g, 20 mmol) at 423–433 K for 5 h. The product was dissolved in water and the solution extracted with chloroform. The chloroform phase was dried over sodium sulfate; the evaporation of the solvent gave well shaped colourless prisms of (I).

### Refinement

Carbon-bound H-atoms were placed in calculated positions (C—H 0.93 to 0.96 Å) and were included in the refinement in the riding model approximation, with *U*_iso_(H) set to 1.2 to 1.5*U*_equiv_(C). In the absence of significant anomalous scattering effects, 985 Friedel pairs were averaged in the final refinement.

### Crystal data

**Table:**

C_11_H_10_N_2_O_2_	*F*(000) = 424
*M**_r_* = 202.21	*D*_x_ = 1.332 Mg m^−^^3^
Monoclinic, *C**c*	Mo *K*α radiation, λ = 0.71073 Å
Hall symbol: C -2yc	Cell parameters from 1361 reflections
*a* = 8.8120 (16) Å	θ = 2.2–21.9°
*b* = 18.215 (3) Å	µ = 0.09 mm^−^^1^
*c* = 7.2094 (10) Å	*T* = 293 K
β = 119.380 (2)°	Prism, colourless
*V* = 1008.4 (3) Å^3^	0.40 × 0.30 × 0.08 mm
*Z* = 4	

### Data collection

**Table:**

Bruker SMART APEX CCD diffractometer	1165 independent reflections
Radiation source: fine-focus sealed tube	897 reflections with *I* > 2σ(*I*)
graphite	*R*_int_ = 0.033
ω scans	θ_max_ = 27.5°, θ_min_ = 2.2°
Absorption correction: multi-scan (*SADABS*; Sheldrick, 1996)	*h* = −11→11
*T*_min_ = 0.889, *T*_max_ = 1.000	*k* = −23→23
4725 measured reflections	*l* = −9→9

### Refinement

**Table:**

Refinement on *F*^2^	Hydrogen site location: inferred from neighbouring sites
Least-squares matrix: full	H-atom parameters constrained
*R*[*F*^2^ > 2σ(*F*^2^)] = 0.032	*w* = 1/[σ^2^(*F*_o_^2^) + (0.0443*P*)^2^ + 0.0614*P*] where *P* = (*F*_o_^2^ + 2*F*_c_^2^)/3
*wR*(*F*^2^) = 0.086	(Δ/σ)_max_ < 0.001
*S* = 1.02	Δρ_max_ = 0.10 e Å^−^^3^
1165 reflections	Δρ_min_ = −0.10 e Å^−^^3^
138 parameters	Extinction correction: *SHELXL97* (Sheldrick, 2008), Fc^*^=kFc[1+0.001xFc^2^λ^3^/sin(2θ)]^-1/4^
2 restraints	Extinction coefficient: 0.016 (3)
Primary atom site location: structure-invariant direct methods	Absolute structure: nd
Secondary atom site location: difference Fourier map	

### Special details

**Table:**

Geometry. All e.s.d.'s (except the e.s.d. in the dihedral angle between two l.s. planes) are estimated using the full covariance matrix. The cell e.s.d.'s are taken into account individually in the estimation of e.s.d.'s in distances, angles and torsion angles; correlations between e.s.d.'s in cell parameters are only used when they are defined by crystal symmetry. An approximate (isotropic) treatment of cell e.s.d.'s is used for estimating e.s.d.'s involving l.s. planes.
Refinement. Refinement of *F*^2^ against ALL reflections. The weighted *R*-factor *wR* and goodness of fit *S* are based on *F*^2^, conventional *R*-factors *R* are based on *F*, with *F* set to zero for negative *F*^2^. The threshold expression of *F*^2^ > σ(*F*^2^) is used only for calculating *R*-factors(gt) *etc*. and is not relevant to the choice of reflections for refinement. *R*-factors based on *F*^2^ are statistically about twice as large as those based on *F*, and *R*- factors based on ALL data will be even larger.

### Fractional atomic coordinates and isotropic or equivalent isotropic displacement parameters (Å^2^)

**Table:**

	*x*	*y*	*z*	*U*_iso_*/*U*_eq_	
O1	0.5000 (2)	0.02433 (8)	0.5000 (3)	0.0629 (5)	
O2	0.7604 (2)	0.21319 (8)	0.3071 (3)	0.0628 (5)	
N1	0.2553 (3)	0.09456 (10)	0.4018 (3)	0.0595 (5)	
N2	0.2520 (3)	−0.03621 (10)	0.4056 (4)	0.0655 (6)	
C1	0.3265 (3)	0.02912 (11)	0.4325 (4)	0.0510 (5)	
C2	0.0840 (4)	0.09409 (16)	0.3359 (5)	0.0756 (8)	
H2	0.0262	0.1387	0.3129	0.091*	
C3	−0.0084 (4)	0.03096 (19)	0.3015 (5)	0.0828 (9)	
H3	−0.1272	0.0314	0.2555	0.099*	
C4	0.0819 (4)	−0.03306 (17)	0.3379 (5)	0.0789 (8)	
H4	0.0213	−0.0770	0.3141	0.095*	
C5	0.5948 (3)	0.09030 (11)	0.5548 (4)	0.0531 (6)	
C6	0.6335 (3)	0.12134 (10)	0.4098 (4)	0.0484 (5)	
H6	0.5954	0.0995	0.2776	0.058*	
C7	0.7304 (3)	0.18570 (11)	0.4623 (3)	0.0488 (5)	
C8	0.7882 (3)	0.21731 (13)	0.6603 (4)	0.0624 (6)	
H8	0.8527	0.2605	0.6970	0.075*	
C9	0.7481 (4)	0.18335 (17)	0.8027 (4)	0.0771 (8)	
H9	0.7870	0.2045	0.9359	0.093*	
C10	0.6533 (3)	0.11978 (16)	0.7543 (4)	0.0697 (7)	
H10	0.6292	0.0973	0.8529	0.084*	
C11	0.8382 (4)	0.28395 (15)	0.3411 (5)	0.0868 (9)	
H11A	0.8443	0.2986	0.2169	0.130*	
H11B	0.9535	0.2823	0.4615	0.130*	
H11C	0.7692	0.3187	0.3676	0.130*	

### Atomic displacement parameters (Å^2^)

**Table:**

	*U*^11^	*U*^22^	*U*^33^	*U*^12^	*U*^13^	*U*^23^
O1	0.0614 (11)	0.0435 (8)	0.0919 (13)	0.0088 (7)	0.0438 (11)	0.0096 (8)
O2	0.0722 (11)	0.0520 (9)	0.0643 (10)	−0.0123 (8)	0.0335 (8)	−0.0019 (8)
N1	0.0551 (11)	0.0530 (11)	0.0691 (12)	0.0075 (9)	0.0294 (10)	−0.0016 (10)
N2	0.0773 (15)	0.0501 (12)	0.0735 (14)	−0.0070 (10)	0.0403 (12)	−0.0005 (10)
C1	0.0553 (14)	0.0481 (12)	0.0565 (14)	0.0034 (10)	0.0329 (12)	0.0045 (10)
C2	0.0570 (16)	0.0763 (17)	0.087 (2)	0.0111 (14)	0.0299 (15)	−0.0078 (15)
C3	0.0562 (17)	0.101 (2)	0.087 (2)	−0.0074 (16)	0.0322 (15)	−0.0199 (17)
C4	0.076 (2)	0.0799 (19)	0.084 (2)	−0.0262 (17)	0.0412 (16)	−0.0136 (15)
C5	0.0468 (12)	0.0451 (12)	0.0666 (14)	0.0094 (9)	0.0273 (11)	0.0057 (10)
C6	0.0480 (12)	0.0415 (10)	0.0535 (12)	0.0036 (9)	0.0231 (10)	−0.0013 (9)
C7	0.0427 (11)	0.0464 (10)	0.0546 (13)	0.0040 (9)	0.0219 (10)	0.0008 (10)
C8	0.0555 (13)	0.0619 (13)	0.0614 (15)	−0.0069 (11)	0.0223 (12)	−0.0156 (12)
C9	0.0779 (19)	0.097 (2)	0.0549 (15)	−0.0069 (16)	0.0315 (14)	−0.0186 (15)
C10	0.0733 (17)	0.0811 (18)	0.0651 (17)	0.0059 (14)	0.0419 (14)	0.0032 (14)
C11	0.103 (2)	0.0589 (16)	0.088 (2)	−0.0223 (14)	0.0389 (19)	0.0037 (14)

### Geometric parameters (Å, °)

**Table:**

O1—C1	1.361 (3)	C5—C6	1.372 (3)
O1—C5	1.405 (3)	C5—C10	1.377 (3)
O2—C7	1.365 (3)	C6—C7	1.389 (3)
O2—C11	1.424 (3)	C6—H6	0.9300
N1—C1	1.314 (3)	C7—C8	1.384 (3)
N1—C2	1.342 (3)	C8—C9	1.384 (4)
N2—C1	1.327 (3)	C8—H8	0.9300
N2—C4	1.330 (4)	C9—C10	1.369 (4)
C2—C3	1.360 (4)	C9—H9	0.9300
C2—H2	0.9300	C10—H10	0.9300
C3—C4	1.363 (4)	C11—H11A	0.9600
C3—H3	0.9300	C11—H11B	0.9600
C4—H4	0.9300	C11—H11C	0.9600
			
C1—O1—C5	117.01 (16)	C5—C6—H6	120.3
C7—O2—C11	117.50 (19)	C7—C6—H6	120.3
C1—N1—C2	114.5 (2)	O2—C7—C8	124.9 (2)
C1—N2—C4	113.7 (2)	O2—C7—C6	115.23 (18)
N1—C1—N2	128.9 (2)	C8—C7—C6	119.9 (2)
N1—C1—O1	118.57 (19)	C7—C8—C9	118.7 (2)
N2—C1—O1	112.54 (19)	C7—C8—H8	120.7
N1—C2—C3	122.6 (3)	C9—C8—H8	120.7
N1—C2—H2	118.7	C10—C9—C8	122.4 (3)
C3—C2—H2	118.7	C10—C9—H9	118.8
C2—C3—C4	116.6 (3)	C8—C9—H9	118.8
C2—C3—H3	121.7	C9—C10—C5	117.7 (2)
C4—C3—H3	121.7	C9—C10—H10	121.2
N2—C4—C3	123.6 (3)	C5—C10—H10	121.2
N2—C4—H4	118.2	O2—C11—H11A	109.5
C3—C4—H4	118.2	O2—C11—H11B	109.5
C6—C5—C10	122.0 (2)	H11A—C11—H11B	109.5
C6—C5—O1	118.3 (2)	O2—C11—H11C	109.5
C10—C5—O1	119.6 (2)	H11A—C11—H11C	109.5
C5—C6—C7	119.3 (2)	H11B—C11—H11C	109.5
			
C2—N1—C1—N2	0.6 (4)	C10—C5—C6—C7	−2.0 (3)
C2—N1—C1—O1	−179.8 (2)	O1—C5—C6—C7	−178.59 (19)
C4—N2—C1—N1	0.1 (4)	C11—O2—C7—C8	−8.0 (3)
C4—N2—C1—O1	−179.5 (2)	C11—O2—C7—C6	171.7 (2)
C5—O1—C1—N1	6.9 (3)	C5—C6—C7—O2	−179.02 (19)
C5—O1—C1—N2	−173.5 (2)	C5—C6—C7—C8	0.7 (3)
C1—N1—C2—C3	−0.7 (4)	O2—C7—C8—C9	180.0 (2)
N1—C2—C3—C4	0.2 (5)	C6—C7—C8—C9	0.3 (3)
C1—N2—C4—C3	−0.8 (4)	C7—C8—C9—C10	−0.1 (4)
C2—C3—C4—N2	0.6 (5)	C8—C9—C10—C5	−1.1 (4)
C1—O1—C5—C6	−100.4 (2)	C6—C5—C10—C9	2.1 (4)
C1—O1—C5—C10	82.8 (3)	O1—C5—C10—C9	178.7 (2)

### Hydrogen-bond geometry (Å, °)

**Table:**

Cg2 is the centroid of the C5–C10 ring.

**Table:**

*D*—H···*A*	*D*—H	H···*A*	*D*···*A*	*D*—H···*A*
C4—H4···Cg2^i^	0.93	2.89	3.710 (4)	148

Symmetry codes: (i) *x*−1, −*y*, *z*−1/2.
